# Some temporal and geographic records of *Abatus* species (Echinoidea, Schizasteridae) in Antarctic and Subantarctic environments

**DOI:** 10.3897/BDJ.14.e202389

**Published:** 2026-10-01

**Authors:** Fernando Moya, Claudia S. Maturana, Sebastian Rosenfeld, Melanie Delleuze, Guillaume Schwob, Camila Neder, Paul Brickle, Thomas Saucède, Léa Cabrol, Karin Gerard, Angie Díaz, Elie Poulin

**Affiliations:** 1 Millenium Institute Biodiversity of Antarctic and Subantarctic Ecosystems (BASE), Santiago, Chile Millenium Institute Biodiversity of Antarctic and Subantarctic Ecosystems (BASE) Santiago Chile; 2 Laboratorio de Ecología Molecular, Departamento de Ciencias Ecológicas, Facultad de Ciencias, Universidad de Chile, Santiago, Chile Laboratorio de Ecología Molecular, Departamento de Ciencias Ecológicas, Facultad de Ciencias, Universidad de Chile Santiago Chile; 3 Cape Horn International Center (CHIC), Puerto Williams, Chile Cape Horn International Center (CHIC) Puerto Williams Chile; 4 Marine Biology Lab, CP160/15, Université Libre de Bruxelles (ULB), Brussels, Belgium Marine Biology Lab, CP160/15, Université Libre de Bruxelles (ULB) Brussels Belgium; 5 Université Claude Bernard Lyon 1, Laboratoire d'Écologie Microbienne, UMR CNRS 5557, UMR INRAE 1418, VetAgro Sup, Research Team “Bacterial Opportunistic Pathogens and Environment” (BPOE), Lyon, France Université Claude Bernard Lyon 1, Laboratoire d'Écologie Microbienne, UMR CNRS 5557, UMR INRAE 1418, VetAgro Sup, Research Team “Bacterial Opportunistic Pathogens and Environment” (BPOE) Lyon France; 6 South Atlantic Environmental Research Institute, Port Stanley, Falkland Islands, United Kingdom South Atlantic Environmental Research Institute Port Stanley, Falkland Islands United Kingdom; 7 School of Biological Sciences (Zoology), University of Aberdeen, Tillydrone Avenue, Aberdeen, Scotland, United Kingdom School of Biological Sciences (Zoology), University of Aberdeen, Tillydrone Avenue Aberdeen, Scotland United Kingdom; 8 Biogeosciences, UMR6282 Université Bourgogne Europe, CNRS, Dijon, France Biogeosciences, UMR6282 Université Bourgogne Europe, CNRS Dijon France; 9 Aix Marseille University, Univ Toulon, CNRS, IRD, Mediterranean Institute of Oceanography (MIO), Marseille, France Aix Marseille University, Univ Toulon, CNRS, IRD, Mediterranean Institute of Oceanography (MIO) Marseille France; 10 Laboratorio de Ecosistemas Marinos Antárticos y Subantárticos, Facultad de Ciencias, Universidad de Magallanes, Punta Arenas, Chile Laboratorio de Ecosistemas Marinos Antárticos y Subantárticos, Facultad de Ciencias, Universidad de Magallanes Punta Arenas Chile; 11 Departamento de Zoología, Facultad de Ciencias Naturales y Oceanográficas, Universidad de Concepción, Concepción, Chile Departamento de Zoología, Facultad de Ciencias Naturales y Oceanográficas, Universidad de Concepción Concepción Chile

**Keywords:** distribution, echinoids, marine invertebrates, occurrence, sea urchins, Southern Ocean, specimen

## Abstract

**Background:**

The study of biodiversity distribution is essential for its conservation. In particular, knowledge gaps concerning the distribution of Antarctic and Subantarctic benthos lead us to undertake research initiatives. Platforms such as GBIF allow us to gather geographical information, standardise it and subsequently analyse it in a reproducible manner. Here, we present a dataset created in GBIF, which shows an occurrence compilation of three species of the genus *Abatus* (Troschel, 1851). The records collated were organised at the specimen level and collected by project from different countries and dates. The aim of this work is to curate these samples and records, contributing to the knowledge of the biogeographical distribution of these three species across three different macrozones (southern Patagonia, Subantarctic islands and Antarctica).

**New information:**

The occurrence dataset presented here has never been released before and is the result of a rigorous compilation and curation of records to document the distribution of species within the genus *Abatus* across the Southern Ocean. We compiled data from field collections and taxonomic research carried out between 2004 and 2024. The dataset includes 27 occurrences of three recognised species (*Abatus agassizii* (Mortensen, 1910); *Abatus cavernosus* (Philippi, 1845); and *Abatus cordatus* (Verrill, 1876)) and two occurrences of *Abatus* identified only at genus level (*Abatus* sp.), which were obtained from multiple locations including the Magellan Strait, Kerguelen Islands, Falkland/Malvinas Islands, South Georgia and South Shetland Islands. All records were georeferenced and curated by experts from the Millennium Institute Biodiversity of Antarctic and Subantarctic Ecosystems (BASE) and associated institutions. The dataset has been published through the Global Biodiversity Information Facility (GBIF) and is accessible under a CC-BY 4.0 licence. This work was supported by various research programmes and contributes to international biodiversity initiatives focused on the Southern Ocean.

## Introduction

Studying species distribution is a central focus of ecology and biogeography as it addresses scientific issues about why species occur where they do, why they are absent from other areas, and the underpinning factors of these patterns (Krebs 2014). Analyses of the geographic distributions of organisms provide insights into biogeographic patterns and help to formulate evolutionary hypotheses (Glasby and Alvarez 1999, Griffiths et al. 2009, Pertierra et al. 2020, González-Wevar et al. 2021, O’Hara et al. 2025). It also allows researchers to evaluate the habitat suitability for species presence and to assess potential range shifts under climate change scenarios or the likelihood of colonisation by invasive species through species distribution models (Newbold et al. 2014, Ukkonen et al. 2014, Bartlett et al. 2020, López‐Farrán et al. 2021, Neder et al. 2024). Furthermore, a species' geographic range is a fundamental criterion for determining its extinction risk, which provides essential information to assess an appropriate conservation status and to develop effective management and protection strategies (IUCN 2025).

In Antarctic and Subantarctic Regions, numerous factors cause spatial variability in species distributions and environmental conditions may differ greatly even between nearby locations, while remaining homogeneous over larger areas (Griffiths 2010). Therefore, understanding the species distribution patterns in these environments helps us interpret biodiversity trends in relation to environmental dynamics and identify priority areas for conservation (Rosenfeld et al. 2022). However, biological sampling in the Southern Ocean has been irregular and significant knowledge gaps persist for geographic areas and depth ranges (Kaiser et al. 2013). Even amongst well-studied groups, sampling tends to be biased; for instance, vertebrates also have far more occurrence records than invertebrates (Griffiths 2010). Echinoids (sea urchins) are one example; they exhibit high diversity, endemism and ecological importance in Antarctic benthic communities (David et al. 2005), but their documented distributions remain fragmented due to uneven sampling across regions and years (Fabri‐Ruiz et al. 2020). Historical specimen collections can help fill these gaps by providing records of species occurrences across broad temporal and spatial scales. These records can contribute to reconstructing distribution ranges and may help evaluate potential changes over time; however, any analysis of temporal changes must account for biases in sampling effort, design and coverage (Pyke and Ehrlich 2010).

*Abatus* is a genus of irregular sea urchins (family Schizasteridae) distributed mainly in the Southern Ocean. These urchins are deposit feeders with an infaunal lifestyle, ingesting surface sediment to feed on detritus and inhabiting the subtidal zone down to approximately 1000 m depth (David et al. 2005). Three species of *Abatus (A. agassizii, A. cavernosus* and *A. cordatus)* are phylogenetically, morphologically and ecologically related (Diaz et al. 2012, David et al. 2016, Delleuze et al. 2024) forming a clade within the genus, sharing descriptive characters and inhabiting shallow-water (subtidal) environments. For many years, these *Abatus* species have been used as model organisms for studying direct development, morphology, biogeography, connectivity, host-associated microbiota and evolutionary patterns (Poulin and Féral 1995, Diaz et al. 2012, Ledoux et al. 2012, Schwob et al. 2020, Pascal et al. 2021, Schwob et al. 2021, Delleuze et al. 2024), as well as sentinels for detecting the biological effects of global change in the Southern Ocean (Arnould-Pétré et al. 2021, Moya et al. 2025). Given their ecological importance and unique life-history traits, improving knowledge of *Abatus* species distributions is crucial for filling information gaps (such as the lack of accessible, standardised and georeferenced occurrences), testing eco-evolutionary hypotheses and enhancing our understanding of Antarctic and Subantarctic biodiversity. In this work, we used specimens from a biological collection gathered during numerous Antarctic and Subantarctic expeditions (2004–2024) to contribute to the knowledge of the distribution of this clade across the Southern Ocean using spatial and temporal records.

## General description

### Purpose

This dataset was compiled as part of the first author's Master's thesis and the principal investigator's ongoing research. The main goal of the dataset is to establish a baseline of the biogeographic distribution of the three *Abatus* species. This baseline complements current research on the biogeography of the genus *Abatus* being carried out at the Laboratory of Molecular Ecology (LEM) at the University of Chile.

## Project description

### Title

Some temporal and geographic records of *Abatus* species (Echinoidea, Schizasteridae) in Antarctic and Subantarctic environments

### Personnel

Fernando Moya, Claudia Maturana, Sebastian Rosenfeld, Melanie Delleuze, Guillaume Schwob, Camila Neder, Paul Brickle, Thomas Saucède, Léa Cabrol, Angie Díaz, Karin Gerard and Elie Poulin

### Study area description

The study area corresponds to shallow coastal waters in the subtidal zone of Antarctic and Subantarctic regions.

### Funding

ANID-Programa Iniciativa Científica Milenio-ICN2021_002, FONDECYT Regular 1161358 and 1211672, IPEV project n°1044 PROTEKER, South Atlantic Environmental Research Institute (SAERI) and Fondecyt Postdoctoral project 3100139.

## Sampling methods

### Study extent

Samples were collected through various projects and expeditions in the Magellan Strait (Patagonia, South America), the Kerguelen Islands (several sites during the Proteker project campaigns 1, 5, 8 and 9), the Falkland/Malvinas Islands, South Georgia (with logistical support of the South Atlantic Environmental Research Institute) and the South Shetland Islands during Antarctic Scientific Expeditions (ECA), supported by the Chilean Antarctic Institute (2004–2024) (Fig. 1).

### Sampling description

To sample the *Abatus* species, two complementary collection techniques were employed: 1) hand-picking in the infralittoral zone at low tide and 2) Self-Contained Underwater Breathing Apparatus (SCUBA) diving to sample subtidal habitat at depths of 1-20 m. Depending on the sampling campaign, collected specimens were transported to research vessels or scientific stations with *in situ* seawater. They were subsequently preserved in 95% ethanol in the onboard or station laboratories.

### Quality control

Through field surveys, accessible sites with specific environmental conditions (sediment type, grain size and protection from waves) were selected for sampling. Within each site, if the subtidal zone was shallow, samples were taken by hand at random points . If the subtidal zone was deep, SCUBA diving surveys were carried out systematically spaced at approximately 100 metres for Falkland/Malvinas Islands, Magellan Strait, South Shetlands Islands and South Georgia sites and 500 metre intervals for Kerguelen sites, ensuring methodical spatial coverage. After sampling, all specimens were preserved in 95% ethanol for both morphological and molecular analyses and were stored at room temperature. The GPS coordinates were recorded for each occurrence to ensure accurate georeferencing. To reduce any taxonomic misidentification, each urchin was revised by experts in the group and identified, based on diagnostic morphological characteristics following literature and the online taxonomic key "Antarctic Echinoidea".

### Step description

The step-by-step methodology details of each project can be obtained from their respective researchers through the provided contact information (email) in the GBIF dataset.

## Geographic coverage

### Description

This dataset encompasses records of *Abatus* species from five main regions across the Southern Ocean (Fig. 1). These include Possession Bay in the Magellan Strait (Patagonia; Fig. 1A); Canache area in the Falkland Islands/Malvinas (Fig. 1B); Stromness Bay in South Georgia (Fig. 1C); Port Aux Français, Fjords des Portes Noires, Anse du Halage, Haute, Port Matha, Ile Suhm and Port Couvreux in Kerguelen (Fig. 1D); and China Cove and Ardley Island in Fildes Bay (King George Island; South Shetland Islands) (Fig. 1E). The subantarctic zone (Patagonia, Kerguelen, Falkland/Malvinas and South Georgia) accounts for 62.1% of the recorded occurrences, whereas the Antarctic zone (South Shetland Islands) contributes the remaining 37.9% of occurrences (Fig. 2).

### Coordinates

−64.876 and −48.935 Latitude; −69.478 and 70.219 Longitude.

## Taxonomic coverage

### Description

This dataset includes three species of the genus *Abatus* (order Spatangoida; family Schizasteridae) of the eleven species currently recognised in this genus (David et al. 2005). Each of the three recorded species is associated with a specific geographic range: *Abatus agassizii* (Fig. 3A) occurs in the South Shetland Islands, *Abatus cordatus* (Fig. 3B) occurs in the Kerguelen Islands (both have the highest number of records in this dataset, with 11 occurrences per species) and *Abatus cavernosus* (Fig. 3C) occurs in the Subantarctic Region of South America (Patagonia), primarily in the eastern Magellan Strait with five occurrences. The dataset also includes one occurrence in the Falkland/Malvinas Islands (Fig. 3D) and one in South Georgia (Fig. 3E), which represent an *Abatus* species that have not been identified due to some overlapping characters (e.g. type of pedicellariae and test shape) which limits a reliable identification.

### Taxa included

**Table:** 

Rank	Scientific Name	Common Name
species	*Abatus agassizii*	heart urchins
species	*Abatus cavernosus*	heart urchins
species	*Abatus cordatus*	heart urchins

## Temporal coverage

**Data range:** 2004-2-17 – 2024-3-12.

### Notes

This dataset includes occurrence records of *Abatus* species from 2004 to 2024. The Antarctic Scientific Expeditions (ECAs) allowed sampling of *A. agassizii* in the Antarctic sector during 2004, 2006, 2009, 2010, 2011, 2017, 2020 and 2024. The PROTEKER campaigns provided samples of *A. cordatus* in different areas of the Kerguelen Islands during 2011, 2019 and 2021. Expeditions to the Magellan Strait during 2022, 2023 and 2024 allowed the collection of the species *A. cavernosus*. Finally, occurrences from South Georgia and the Falkland/Malvinas Islands were recorded only in 2021 and 2022, respectively (*Fig. 4*).

## Collection data

### Collection name

Colección de Invertebrados del Laboratorio de Ecología Molecular de la Universidad de Chile 

### Collection identifier

urn:BASE:LEM:Inv:Ech:Echi:Aba 

### Specimen preservation method

95% Ethanol.

## Usage licence

### Usage licence

Other

### IP rights notes

This work is licensed under a Creative Commons Attribution (CC-BY 4.0) Licence.

## Data resources

### Data package title

Some temporal and geographic records of *Abatus* species (Echinoidea, Schizasteridae) in Antarctic and Subantarctic environments

### Resource link

https://doi.org/10.15468/gmsy5y

### Alternative identifiers

https://www.gbif.org/dataset/57d14cdb-47c9-49ac-99f9-d0796c8eec80

### Number of data sets

1

### Data set 1.

#### Data set name

Some temporal and geographic records of *Abatus* species (Echinoidea, Schizasteridae) in Antarctic and Subantarctic environments 

#### Data format

Darwin Core

#### Download URL

https://gbif-chile.mma.gob.cl/ipt/archive.do?r=abatus_so

#### Description

The data presented here are available through the Global Biodiversity Information Facility (Moya et al. 2024b). The database presents a compilation of records of three species of the genus *Abatus* and some samples not identified at species level. All samples are organised at the specimen level and collected by projects from different countries and dates.

**Data set 1.** 

Column label	Column description
occurrenceID	Unique identifier for each occurrence per taxa.
basisOfRecord	The specific nature of the data record. We used the recommended best practice of one of the Darwin Core classes.
institutionCode	Unique identifier for the institution having custody of the object(s) or information referred to in the record.
collectionCode	The code identifying the collection from which the record was derived.
catalogNumber	An unique identifier for the record within the dataset.
type	The nature of the resource or record. For example, physical object, image, sound or event.
language	The language of the description of different localities.
collectionID	The identifier for the collection or dataset from which the record was derived.
occurrenceRemarks	Additional comments, notes or information on the biologicalrecord.
recordedBy	A person or a list of names of people responsible for recording the original Occurrence.
individualCount	The number of individuals present at the time of the Occurrence when it is countable.
sex	The sex of the biological individual(s) represented in the dwc:Occurrence.
lifeStage	The age class or life stage of the dwc:Organism(s) at the time the dwc:Occurrence was recorded.
preparations	A list concatenated and separated of preparations and preservation methods for the specimen.
eventID	An identifier for the set of information associated with a dwc:Event (something that occurs at a place and time).
samplingProtocol	The methods or protocols used during a dwc:Event.
samplingEffort	The amount of effort expended during a dwc:Event. In this case, time.
eventDate	The date-time when the event was recorded. We used best practice using the ISO 8601:1:2019.
eventTime	The time or interval during which a dwc:Event occurred.
year	The four-digit year in which the Occurrence was recorded, according to the Common Era Calendar.
month	The integer month in which the Occurrence was recorded.
day	The integer day of the month on which the dwc:Event occurred.
verbatimEventDate	The verbatim original representation of the date and time information for a dwc:Event.
habitat	A category or description of the habitat in which the dwc:Event occurred.
fieldNotes	Text of notes taken in the field about the dwc:Event.
continent	The name of the continent in which the dcterms:Location occurs.
island	The name of the island which the Location occurs.
country	The name of the country or major administrative unit in which the Location occurs.
countryCode	The standard code for the country in which the Location occurs following the best practice using an ISO 3166-1-alpha-2 country code.
stateProvince	The name of the next smaller administrative region than country.
locality	The specific description of the place in which the collection was made.
verbatimDepth	The original description of the depth below the local surface.
minimumDepthInMetres	The lesser depth below the local surface in metres.
maximumDepthInMetres	The greater depth below the local surface in metres.
verbatimLatitude	The verbatim original latitude of the dcterms:Location.
verbatimLongitude	The verbatim original longitude of the dcterms:Location.
verbatimCoordinateSystem	The verbatim original spatial coordinates of the Location.
decimalLatitude	The geographic latitude in decimal degrees of the geographic centre of a Location. Positive values are north of the Equator, negative values are south of it. Legal values lie between -90 and 90, inclusive.
decimalLongitude	The geographic longitude in decimal degrees of the geographic centre of a Location. Positive values are east of the Greenwich Meridian, negative values are west of it. Legal values lie between -180 and 180, inclusive.
geodeticDatum	The geodetic datum upon which the geographic coordinates given in decimalLatitude and decimalLongitude are based.
coordinateUncertaintyInMetres	The horizontal distance in metres from the given decimalLatitude and decimalLongitude describing the smallest circle containing the whole of the Location.
coordinatePrecision	A decimal representation of the precision of the coordinates given in the dwc:decimalLatitude and dwc:decimalLongitude.
georeferencedBy	A person or a list concatenated and separated of names of people who determined the georeference for the Location.
identifiedBy	A person or a list of names of people responsible for the taxonomic identification of the samples or records.
dateIdentified	The date on which the subject was determined as representing the dwc:Taxon.
taxonID	An identifier for the set of dwc:Taxon information.
scientificName	The full scientific name in the lowest level taxonomic rank that was determined and the author of the first description.
kingdom	The full scientific name of the kingdom in which the taxon is classified.
phylum	The full scientific name of the phylum in which the taxon is classified.
class	The full scientific name of the class in which the taxon is classified.
order	The full scientific name of the order in which the taxon is classified.
family	The full scientific name of the family in which the taxon is classified.
genus	The full scientific name of the genus in which the taxon is classified.
specificEpithet	The name of species epithet of the scientificName.
taxonRank	The taxonomic rank of the most specific name in the scientificName.
scientificNameAuthorship	The authorship information for the scientificName formatted according to the conventions of the applicable nomenclaturalCode.

## Additional information

### Discussion

This work consolidates two decades (2004–2024) of georeferenced occurrences for three *Abatus* species across five sectors of the Southern Ocean from various Antarctic and Subantarctic research campaigns, providing a brief, but explicit spatio-temporal baseline for future ecological, biogeographic and conservation analyses (Moya et al. 2024a). While unequal sampling does not allow us to infer abundance trends, the repeated detections of *A. cordatus* in the Kerguelen Islands and *A. agassizii* in Fildes Bay (King George Island) in multiple expeditions throughout different years show a persistent local presence, suggesting a temporary maintenance of the patches in those sites, consistent with the species' short generation times (around 5 years) and brooding life history (Mesphoulhé and David 1992). Notably, *A. agassizii* appears highly localised within the South Shetlands/Antarctic Peninsula Region, where it is most reliably encountered in the protected, ice-free embayments of Fildes Bay. Conversely, *A. cordatus* is endemic to the Kerguelen Plateau and widespread in shallow soft-bottom habitats there. These patterns align with prior work emphasising the restricted ranges of *A. agassizii* and the endemism of *A. cordatus* in the Kerguelen Islands (Mesphoulhé and David 1992, Palma et al. 2006, Diaz et al. 2012, Arnould-Pétré et al. 2021).

However, unresolved phylogenetic relationships and morphological similarities amongst *Abatus* species have led to erroneous identifications and problems in the taxonomic accuracy within this genus (David et al. 2016, GBIF 2023), limiting the accurate mapping of species distribution ranges. For instance, records from the Falkland/Malvinas Islands, which typically share fauna with Patagonia (Koubbi et al. 2014), highlight taxonomic uncertainty by documenting the presence of *A. agassizii* or potentially *A. philippii (GBIF 2023)*, instead of the expected *A. cavernosus*, which is found in the Magellan Strait as well as in locations on the southern coast of Argentina (e.g. Puerto Deseado; see Gil et al. (2009), Gil et al. (2020)). Similarly, published syntheses of the Scotia Arc report occurrences of multiple species, such as *A. agassizii, A. bidens*, *A. cavernosus* and *A. philippii* (David et al. 2016, GBIF 2023). In Antarctic areas (South Shetland and Antarctic Peninsula), problems between species are evident as well, such as *A. cavernosus* and *A. bidens* for the sector of Maritime Antarctica (GBIF 2023). These discrepancies show the importance of accurate occurrence data and robust taxonomic resolution and specimen-vouchered datasets and integrative taxonomy (targeted barcoding, diagnostic morphology and ecological specificity) are most valuable for these purposes. We therefore flag Falkland/Malvinas/South Georgia *Abatus* occurrences as priorities for DNA sequencing and standardised re-identification to resolve whether they belong to *A. cavernosus*, *A. philippii* or an undescribed unit.

The life-history of *Abatus* (direct development, maternal brooding and absence of pelagic larvae) constrains dispersal and gene flow, favouring fine-scale spatial genetic structure and demographic sensitivity to habitat fragmentation (Poulin and Féral 1996, Ledoux et al. 2012). Empirical studies document a long brooding cycle, limited mobility and significant micro-spatial genetic variation in *Abatus* species. Additionally, individual-based simulations indicate that these populations are vulnerable to local disturbances, as supported by multiple studies (Ledoux et al. 2012, Maturana et al. 2016, Fabri‐Ruiz et al. 2020, Arnould-Pétré et al. 2021, Pascal et al. 2021, Delleuze et al. 2024). Recent morphological and microbiological analyses further confirm this fragility, especially in response to metal contamination (Moya et al. 2025). Our multi-campaign occurrence series, thus, offers a needed baseline to detect future range contractions or shifts under environmental change, especially for brooders whose realised dispersal is intrinsically low (Schatt and Feral 1991, Arnould-Pétré et al. 2021).

Methodologically, open biodiversity aggregators (OBIS, GBIF) are indispensable to contextualise Southern Ocean biodiversity, but exhibit strong spatial, temporal and taxonomic biases due to remoteness, logistics and route-based sampling. We therefore advocate routine occurrence-data QA/QC (e.g. *CoordinateCleaner* pipelines for georeferencing artefacts; depth/land–sea mismatch checks and institutional centroid filters) before downstream analyses (e.g. Species Distribution Models, range metrics). At the Southern Ocean scale, Species Distribution Models for benthic taxa should be constructed with careful bias correction and environmental realism (substrate, bathymetry, near-bottom climatologies) (Zizka et al. 2019, Bonnet-Lebrun et al. 2023), as recent echinoid-focused assessments caution that model robustness otherwise suffers (Fabri‐Ruiz et al. 2020). Our curated, vouchered occurrences provide a high-quality backbone for such analyses, while making explicit where data gaps remain. From a conservation standpoint, shallow Antarctic and Subantarctic soft-bottom communities are exposed to multiple, interacting drivers (ocean warming and marine heatwaves, sea-ice and iceberg-scour dynamics, acidification, pollution and fisheries) whose effects are magnified for brooders with limited dispersal and narrow depth/temperature envelopes. Building on our baseline, future assessments should quantify Extent of Occurrence (EOO) and Area of Occupancy (AOO) following current IUCN Red List guidance, explicitly accounting for sampling bias and taxonomic uncertainty (IUCN 2025). Region-specific ecoregionalisations, based on echinoid distributions, can help translate occurrence baselines into spatial priorities (e.g. Marine protected areas design/updates) under Intergovernmental Panel on Climate Change (IPCC) consistent scenarios.

Taken together, our dataset: (i) documents persistent populations at key localities for *A. cordatus* and *A. agassizii*; (ii) highlights taxonomic uncertainty in the Falkland/Malvinas Islands and South Georgia where integrative work is needed; (iii) provides curated inputs for bias-aware SDMs and future monitoring; and (iv) establishes a reproducible baseline against which to test hypotheses about brooder vulnerability, range dynamics and conservation status across the Southern Ocean (Griffiths 2010). Therefore, this dataset not only addresses existing knowledge gaps, but also establishes a valuable baseline for future biogeographic, ecological and genetic studies aimed at understanding the resilience and evolutionary potential of *Abatus* species in the face of global environmental change.

## Figures and Tables

**Figure 1.**
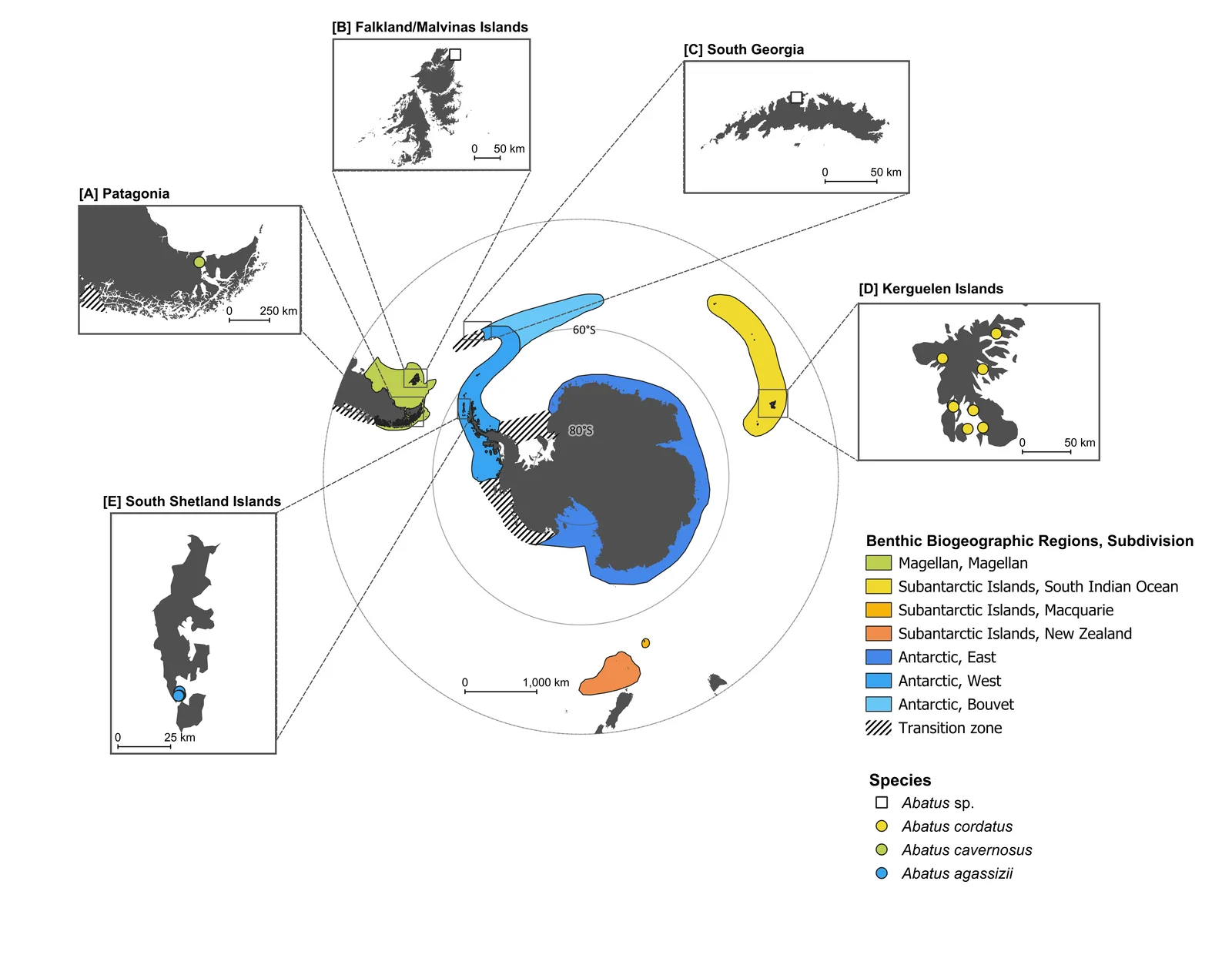
Distribution of *Abatus* occurrences in the Antarctic and Subantarctic zones (Koubbi et al. 2014). Coloured dots represent occurrence records for each species: *Abatus* sp. (white squares), *Abatus cordatus* (yellow circles), *Abatus cavernosus* (green circles) and *Abatus agassizii* (blue circles). Coloured polygons represent the benthic biogeographic regions following Koubbi et al. (2014), including the transition zones (hatched areas). Inset maps show detailed views of specific localities where occurrences were recorded: **[A]** Patagonia, **[B]** Falkland/Malvinas Islands, **[C]** South Georgia, **[D]** Kerguelen Islands and **[E]** South Shetland Islands.

**Figure 2.**
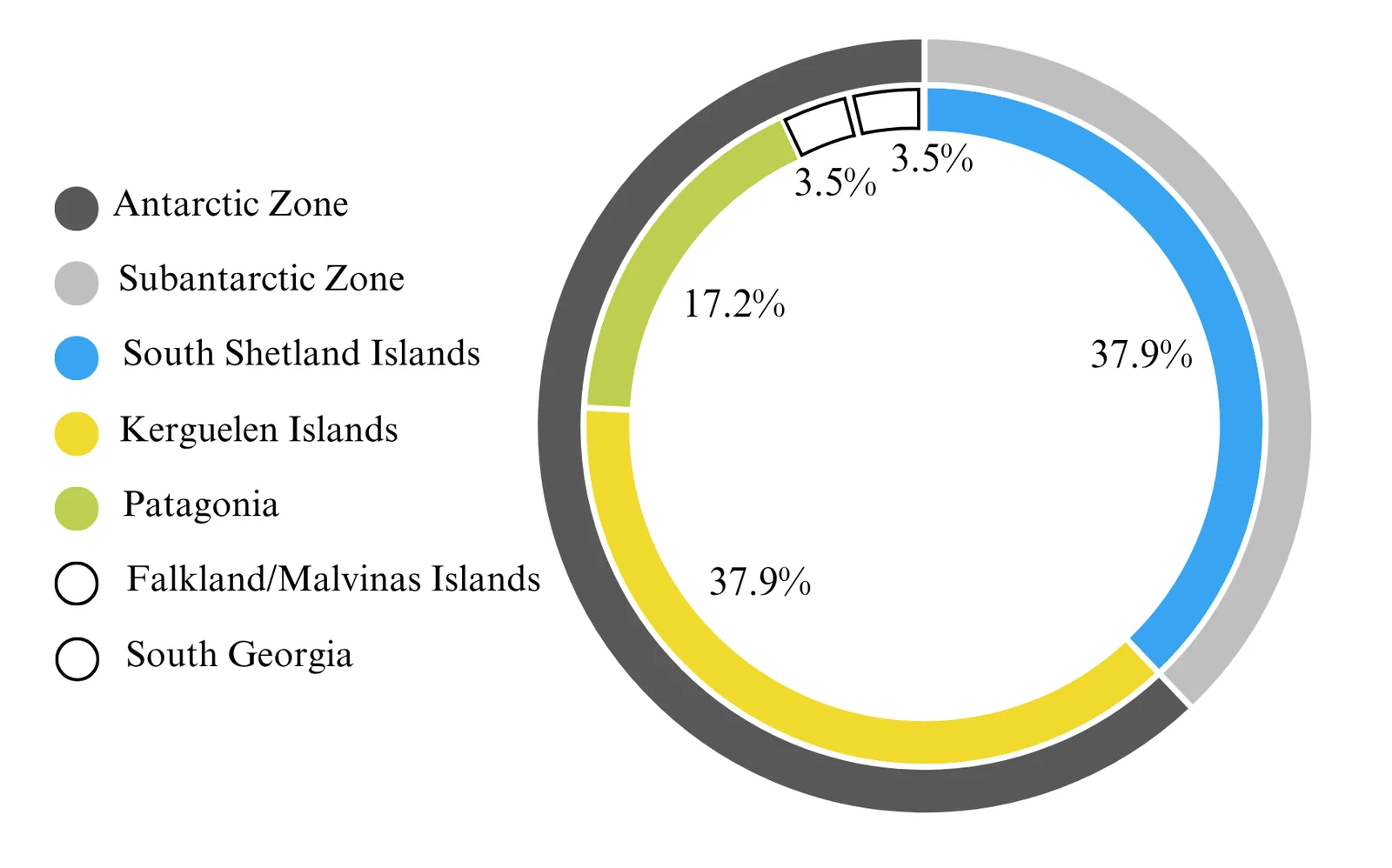
Percentage of occurrences at each sampling site. The outer ring shows the difference between the Antarctic and Subantarctic zones and the inner ring differentiates the occurrences by specific sectors.

**Figure 3.**
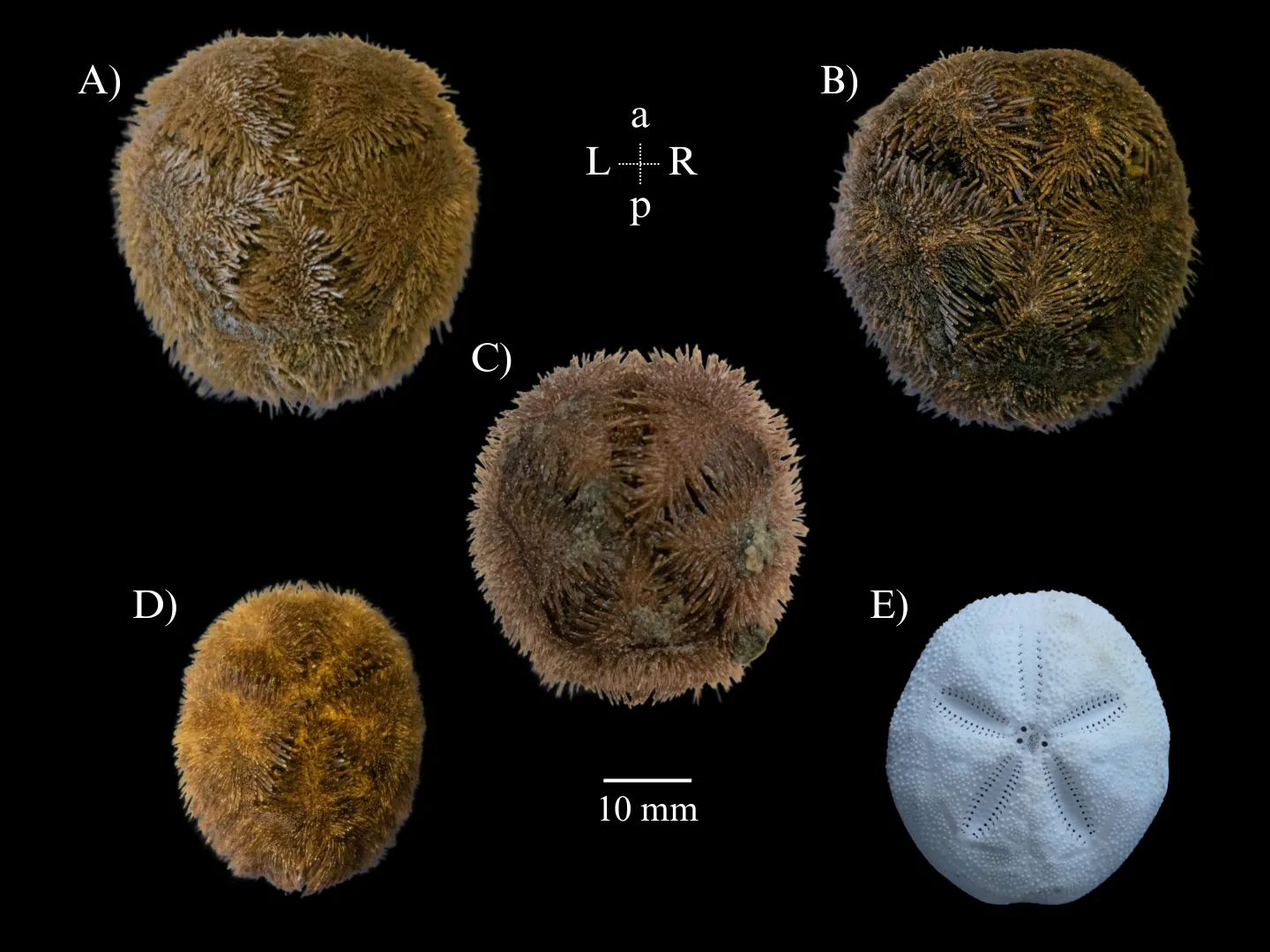
Dorsal (aboral) views of *Abatus* species recorded in this study. **[A]** *Abatus agassizii* from maritime Antarctica; **[B]** *Abatus cordatus* from Kerguelen Islands; **[C]** *Abatus cavernosus* from Patagonia; **[D]** *Abatus* sp. from Falkland/Malvinas Islands; and **[E]** *Abatus* sp. from South Georgia with its test exposed (cleaned). Anterior (a), posterior (p), left side (L) and right side (R) of all the individuals are shown.

**Figure 4.**
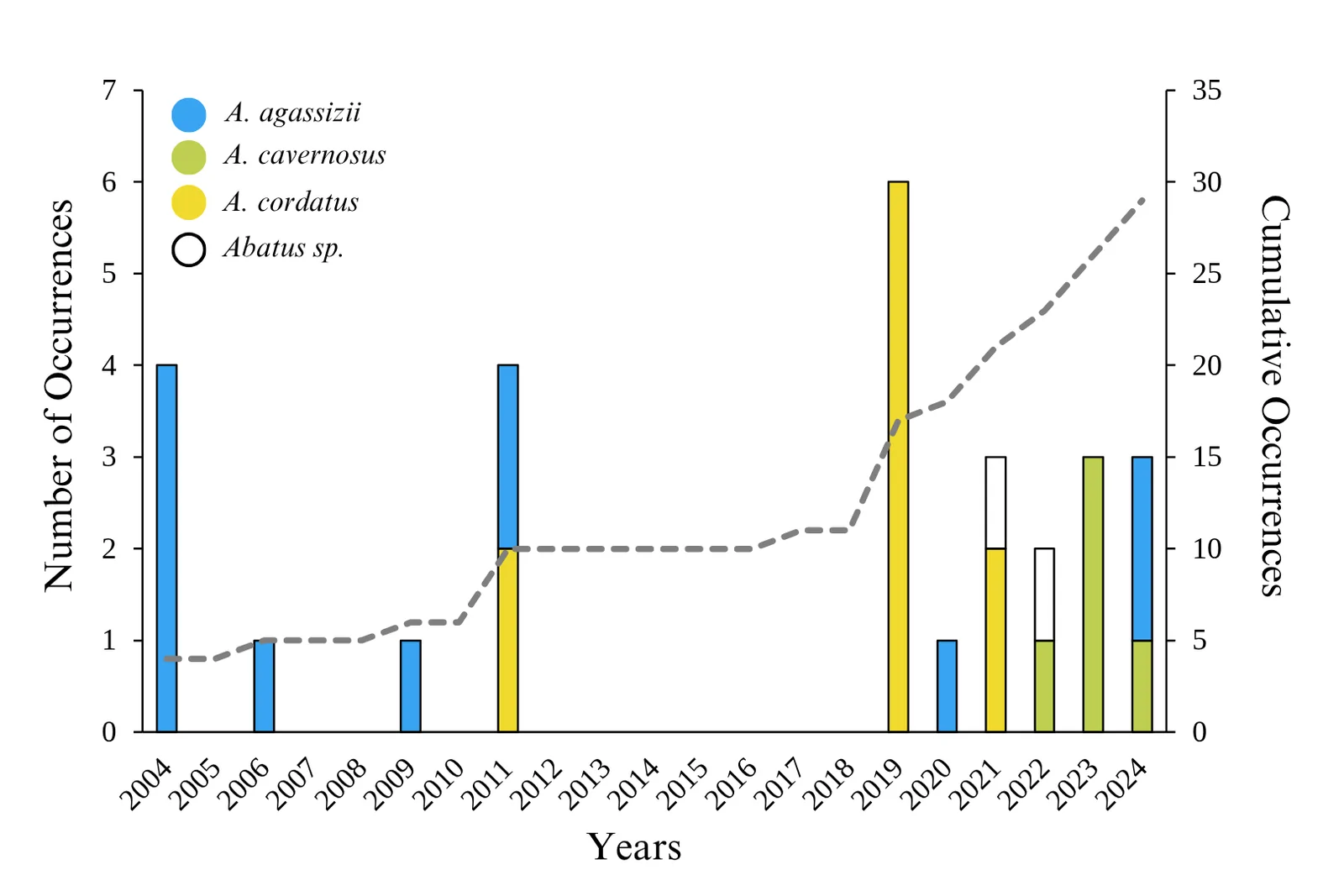
Number of occurrence records per species between 2004 and 2024. The bars represent the annual number of occurrence records for each *Abatus* species (left y-axis), colour-coded according to the *Abatus* species. The dashed grey line indicates the cumulative occurrences across all species over time (right y-axis). These temporal patterns reflect the availability of records within the dataset and are highly influenced by uneven historical sampling effort amongst years.
